# Formation mechanism of axial macrosegregation of primary phases induced by a static magnetic field during directional solidification

**DOI:** 10.1038/srep45834

**Published:** 2017-04-03

**Authors:** Xi Li, Yves Fautrelle, Zhongming Ren, Rene Moreau

**Affiliations:** 1 State Key Laboratory of Advanced Special Steels, Shanghai University, Shanghai, 200072, P. R. China; 2 SIMAP-EPM-Madylam/CNRS, Enshmg, BP 95, 38402, St Martin d’Heres Cedex, France

## Abstract

Understanding the macrosegregation formed by applying magnetic fields is of high commercial importance. This work investigates how static magnetic fields control the solute and primary phase distributions in four directionally solidified alloys (i.e., Al-Cu, Al-Si, Al-Ni and Zn-Cu alloys). Experimental results demonstrate that significant axial macrosegregation of the solute and primary phases (i.e., Al_2_Cu, Si, Al_3_Ni and Zn_5_Cu phases) occurs at the initial solidification stage of the samples. This finding is accompanied by two interface transitions in the mushy zone: quasi planar → sloping → quasi planar. The amplitude of the macrosegregation of the primary phases under the magnetic field is related to the magnetic field intensity, temperature gradient and growth speed. The corresponding numerical simulations present a unidirectional thermoelectric (TE) magnetic convection pattern in the mushy zone as a consequence of the interaction between the magnetic field and TE current. Furthermore, a model is proposed to explain the peculiar macrosegregation phenomenon by considering the effect of the forced TE magnetic convection on the solute distribution. The present study not only offers a new approach to control the solute distribution by applying a static magnetic field but also facilitates the understanding of crystal growth in the solute that is controlled by the static magnetic field during directional solidification.

Applied magnetic fields have the ability to modify significantly the solidification process of alloys in regards to the grain size, crystal growth, and the solute or inclusion distribution^1^,^2^,^3^,^4^,^5^,^6^,^7^,^8^,^9^,^10^. As is well-known, static magnetic fields are capable of damping flow and enhancing the growth of columnar dendrite^11^,^12^. Recent experimental and theoretical works have shown that the TE magnetic effect also plays a key role in affecting the solidification structure during directional solidification under magnetic fields^13^,^14^,^15^. TE magnetic convection in the liquid causes the formation of freckle segregation and TE magnetic force acting on the solid destabilizes the stability of the solid/liquid interface during directional solidification.

It is well-known that controlling the macrosegregation of alloy ingots is of high commercial importance due to the inhomogeneous mechanical property of the product. However, so far, there is a lack of knowledge on the effect of static magnetic fields on the axial macrosegregation of alloys during directional solidification. The present work investigates the effect of static transverse magnetic fields on the distributions of primary phases and solute in four directionally solidified alloys (i.e., Al-Cu, Al-Si, Al-Ni and Zn-Cu alloys). It is observed that significant macrosegregation of the primary phases (i.e., α-Al, Al_2_Cu, Al_3_Ni and Zn_5_Cu phases) is formed at the initial solidification stage of the samples. Furthermore, a model is proposed to explain the peculiar macrosegregation phenomenon during directional solidification under a transverse magnetic field. This work not only presents a new approach to control the solute distribution by applying a static magnetic field but also facilitates the understanding of crystal growth in the solute which is controlled by the static magnetic field during directional solidification.

## Description of the Experimental Device

Raw ingots with a diameter of 100 mm in were prepared in an induction furnace. The samples for directional solidification with a diameter of 3 mm and length of 180 mm were electro-discharge machined from the raw ingots and enveloped in high purity corundum tubes with an inner diameter of 3 mm and length of 200 mm. A schematic view of the directional solidification apparatus under a transverse magnetic field was shown in ref. 16. It consisted of a direct current electromagnet, a Bridgman furnace and a growth velocity and temperature controller. The direct current electromagnet can produce a transverse static magnetic field up to 1.0 T. The temperature in the furnace was controlled by a Pt/6Rh-Pt/30Rh thermocouple inserted in a pure alumina tube. During the directional solidification, the samples in the crucibles were melted and maintained for 30 min and later solidified in the Bridgman apparatus by pulling the crucible assembly at various velocities with and without the transverse magnetic field. At the end of the experiments, the crucibles were quickly dropped into a Ga-In-Sn metal to obtain the microstructure of the solid/liquid interface. The microstructures were examined in the etched condition by optical microscopy.

## Experimental Results

### Primary phase distribution

**Figure 1:**
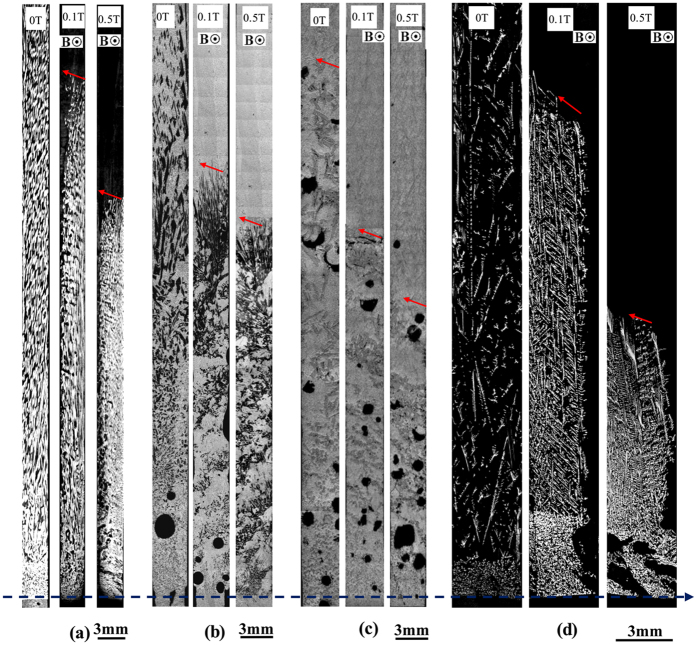
The effect of a transverse magnetic field on the axial macrosegregation of the primary phases during directional solidification. (**a**) Al-40 wt.%Cu alloys, 5 μm/s; (**b**) Al-12 wt.%Ni alloys, 2 μm/s; (**c**) Al-21 wt.%Si alloys, 2 μm/s; (**d**) Zn-2.2 wt.%Cu alloys, 5 μm/s. Red arrows marking the interface of the primary phases and blue lines showing the initial solidification interface.

**Figure 2:**
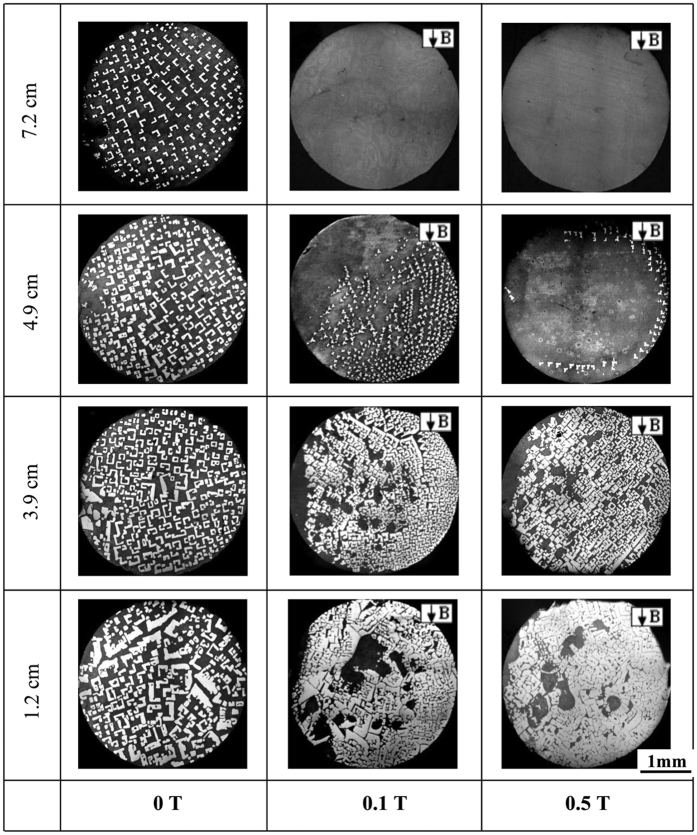
Transverse structures at different positions from the initial solidification interface in directionally solidified Al-40 wt.%Cu alloy at a growth speed of 2 μm/s under various magnetic fields.

**Figure 3:**
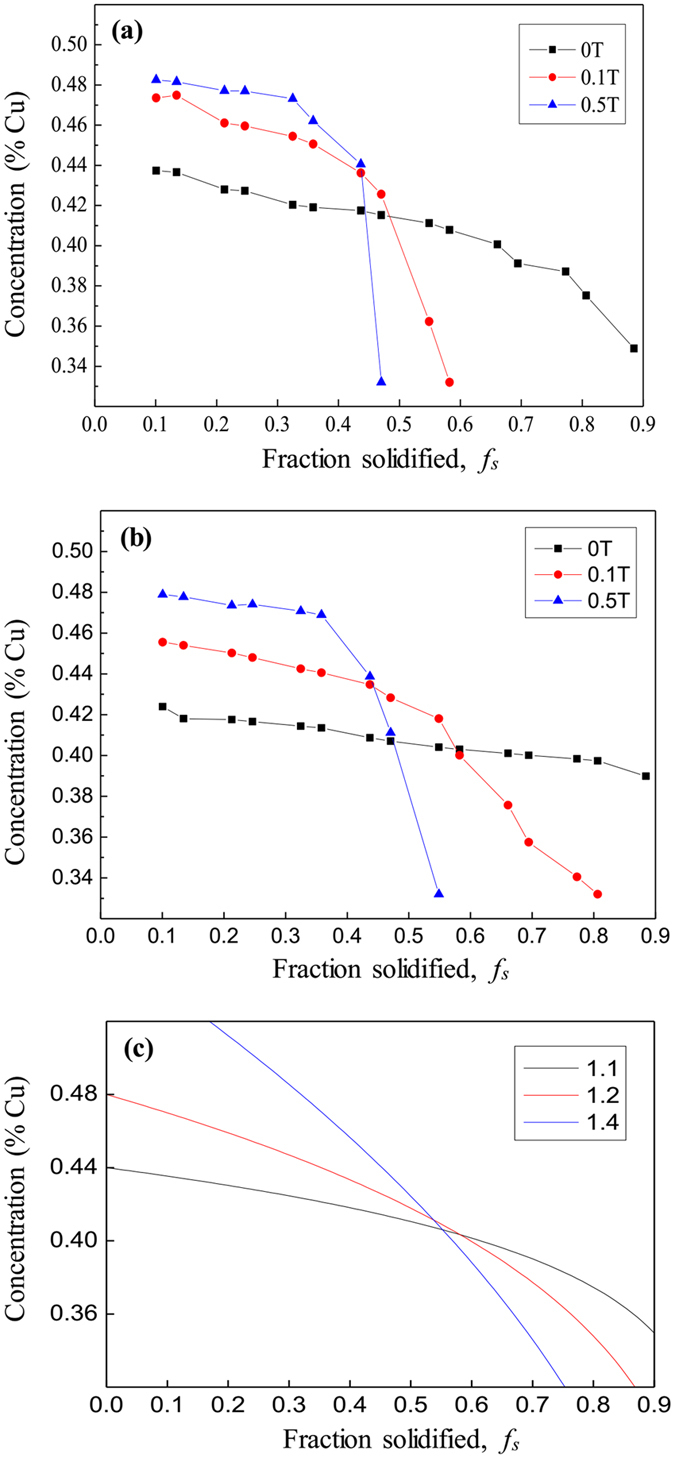
Distribution of concentration along an axial direction as the solidified fraction in the Al-40 wt.%Cu alloys directionally solidified under various magnetic field intensities at 2 μm/s (**a**) and 5 μm/s (**b**,**c**) theoretical Scheil curves in the Al-40 wt.%Cu alloy under various *k*_*e*_values.

Figure 1 shows the longitudinal macrostructures in four directionally solidified hypereutectic alloys (i.e., Al-Cu, Al-Si, Al-Ni and Zn-Cu alloys) with and without the magnetic field. One can notice that the applied magnetic field generates the modification and enrichment of the primary phases at the bottom of the samples. Moreover, such enrichment of the primary phases is enhanced by the magnetic field. The area percentage of the primary Al_3_Ni phases in the Al-12 wt.%Ni alloys directionally solidified at a growth speed of 3 μm/s and a temperature gradient of 60 K/cm under various magnetic fields is shown in Table 1. Figure 2 shows the transverse microstructures in directionally solidified Al-40 wt.%Cu alloy at different positions with and without the magnetic field. Comparison of the solidification structures with and without the magnetic field shows a significant enrichment of the primary Al_2_Cu phases at the bottom of the sample. The primary Al_2_Cu phases disappear when the growth length increases to approximately 6 cm and 5 cm under 0.1 T and 0.5 T magnetic fields, respectively. Figure 3 shows the distribution of the Cu content in directionally solidified Al-40 wt.%Cu alloys at 2 μm/s and 5 μm/s with and without the magnetic field. Comparison of the distribution of the Cu solute with and without the magnetic field shows that the Cu content decreases more quickly as the growth length increases under the magnetic field. The above phenomena become stronger with the increase of the magnetic field and weaker with the increase of the growth speed (see Fig. 3(a) and (b)). Moreover, the theoretical Scheil curves of the Al-40 wt.%Cu hypereutectic alloy with various effective equilibrium partition coefficients (*k*_*e*_) are calculated as follow:


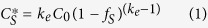


where 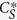 is the solid composition, *k*_*e*_ is the effective equilibrium partition coefficient, *C*_0_ is the average composition of the alloy, and *f*_*s*_ is the phase fraction of the solid. Figure 3(c) shows the theoretical Scheil curves of the Al-40 wt.%Cu alloy with various *k*_*e*_ values (i.e., 1.1, 1.2 and 1.4). Here, the macrosegregation increases with the increase of the *k*_*e*_value. This implies that the applied magnetic field may increase the *k*_*e*_value during directional solidification.

**Figure 4:**
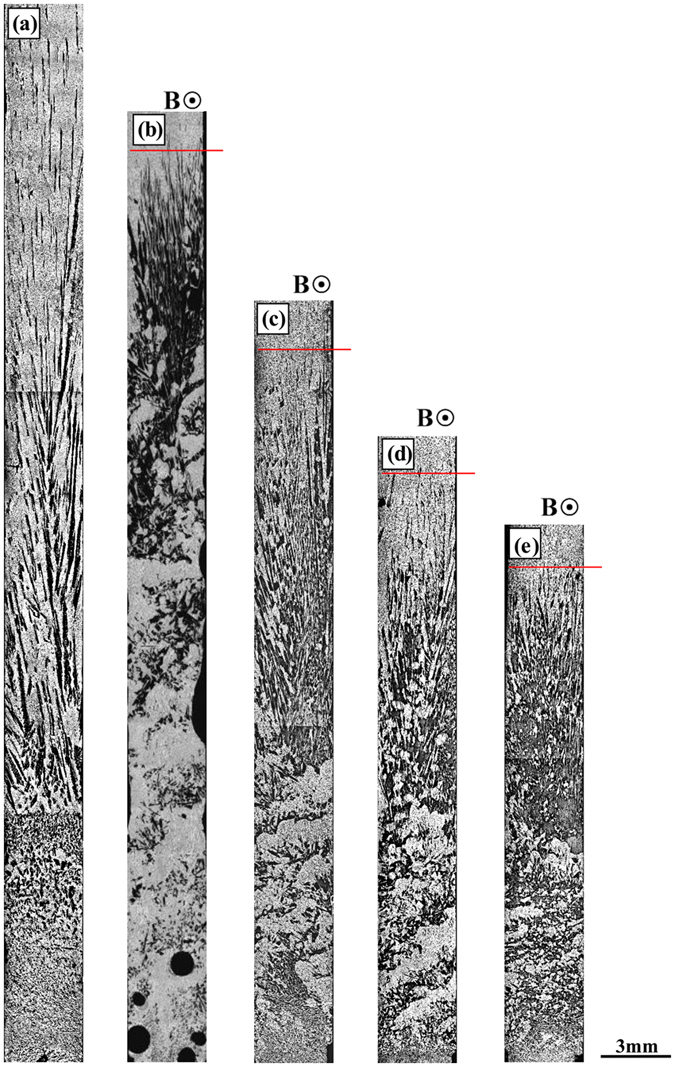
The effect of the temperature gradient on the distribution of the primary Al3Ni phases in the directionally solidified Al-12 wt.% Ni alloy at a growth speed of 5 μm/s under a transverse magnetic field: (**a**) 0 T, 120 K/cm; (**b**) 0.1 T, 60 K/cm; (**c**) 0.1 T, 90 K/cm; (**d**) 0.1 T, 120 K/cm; (**e**) 0.1 T, 150 K/cm. Red lines marked the primary phase interface.

**Figure 5:**
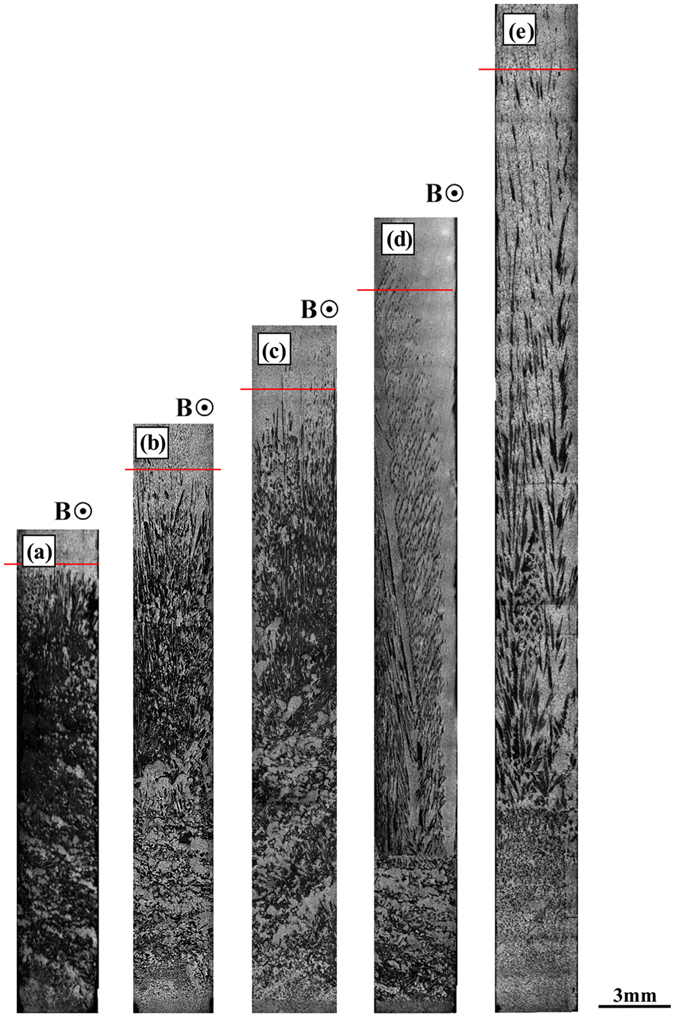
The effect of the growth speed on the distribution of the primary Al3Ni phase in directionally solidified Al-12 wt.% Ni alloy under a transverse magnetic field: (**a**) 0.5 T, 2 μm/s; (**b**) 0.5 T, 5 μm/s; (**c**) 0.5 T, 10 μm/s; (**d**) 0.5 T, 20 μm/s; (**e**) 0 T, 2 μm/s.

Furthermore, the effect of the temperature gradient and growth speed on the distribution of the primary phases during directional solidification under the magnetic field is studied. Figure 4 shows the longitudinal structure in directionally solidified Al-12 wt.%Ni alloy at a growth speed of 2 μm/s and various temperature gradients under a 0.1 T magnetic field. The region length of the primary phase decreases with the increase of the temperature gradient. Figure 5 shows the longitudinal structure in directionally solidified Al-12 wt.%Ni alloys at various growth speeds with and without a 0.5 T magnetic field. With the increase of the growth speed, the effect of the magnetic field on the macrosegregation is weakened. This implies that the temperature gradient and growth speed play important roles in the effect of the magnetic field on the macrosegregation.

### Interface transition in the mushy zone

**Figure 6:**
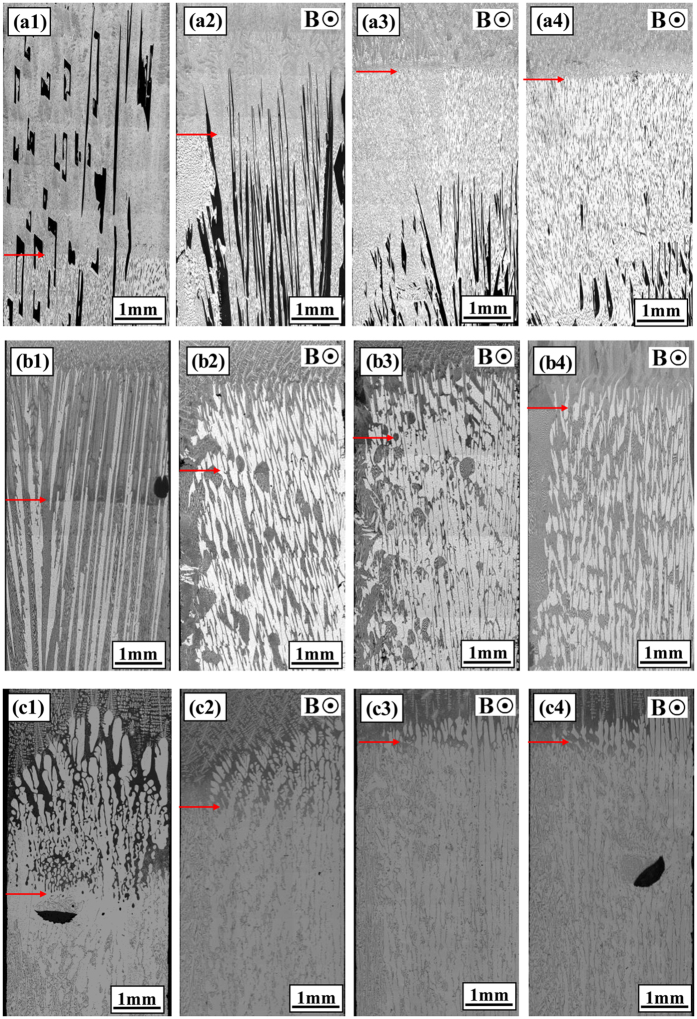
Quenched solid/liquid interface in directionally solidified Al-12 wt.%Ni, Al-40 wt.%Cu and Al-7 wt.%Si alloys after growing a certain distance: (**a**) Al-12 wt.%Ni alloy, 5 μm/s; (**b**) Al-40 wt.%Cu alloy, 5 μm/s; (**c**) Al-7 wt.%Si alloy, 2 μm/s. Red arrows marked the eutectic interface. (a1)-(c1) 0 T; (a2)-(c2) 0.1 T; (a3)-(c3) 0.3 T; (a4)-(c4) 0.5 T.

**Figure 7:**
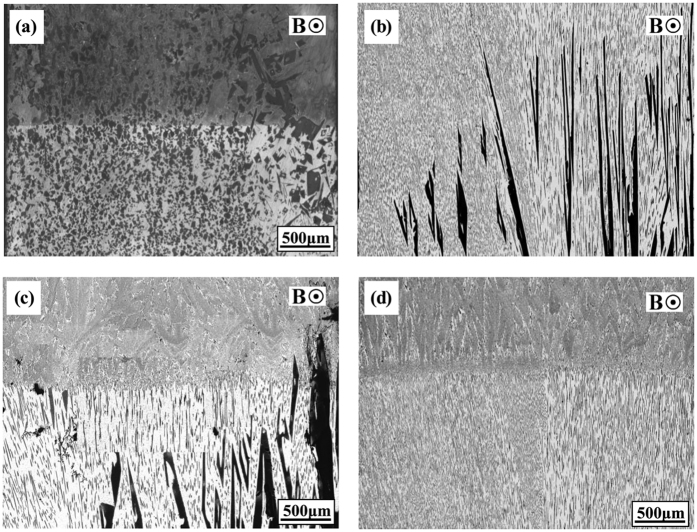
Transitions of the mushy zone in the longitudinal section of the Al-12 wt.%Ni alloys directionally solidified at a growth speed of 5 μm/s under a magnetic field of 0.3 T: growth length at (**a**) 0 cm; (**b**) 2 cm (quenched); (**c**) 3 cm (quenched); (**d**) 5 cm (quenched).

Figure 6 shows the quenched solid/liquid interface in the directionally solidified Al-based alloys (Al-Cu, Al-Si and Al-Ni alloys) after growing a certain distance. The magnetic field induces the formation of the sloping interface and decrease of the mushy zone length. Moreover, it is found that the application of the magnetic field causes the disappearance of the primary phases with the increase of the growth length. Figure 7 shows the evolution of the mushy zone in directionally solidified Al-12 wt.%Ni alloys at 5 μm/s under a 0.3 T magnetic field. Two transitions of the mushy zone interface can be found, i.e., quasi-planar → sloping → quasi-planar, during the primary Al_3_Ni segregation process under a transverse magnetic field. At the initial stage of solidification, a clear horizontal line is observed between the original part and the remelted part in the sample (see Fig. 7(a)). This implies that a quasi-planar interface in the mushy zone is formed before imposing the magnetic field. Figure 7(b) and (c) show the quenched structures at growth lengths of 1 cm and 2 cm, respectively. A sloping solid/liquid interface constructed with the primary phases is observed in the mushy zone. A reproduced quasi-planar interface appears in the quenched structure at a growth length of 5 cm as seen in Fig. 7(d).

## Computation Description and Results

The numerical simulations are performed based on the present experimental results. In this study, the numerical simulations only emphasize the influence of the external magnetic field on the fluid flow and do not consider the heat transfer or the solidification process. To calculate the TE currents at the solid/liquid interface during directional solidification, a complementary term is added to Ohm’s law:


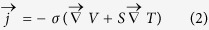


where *V* is the electric scalar potential, *S* is the absolute thermoelectric power, and 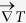 denotes the temperature gradient. The second term on the right hand side of this equation is the contribution of the TE current. The TE current density satisfies the continuity equation:


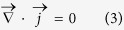


**Figure 8:**
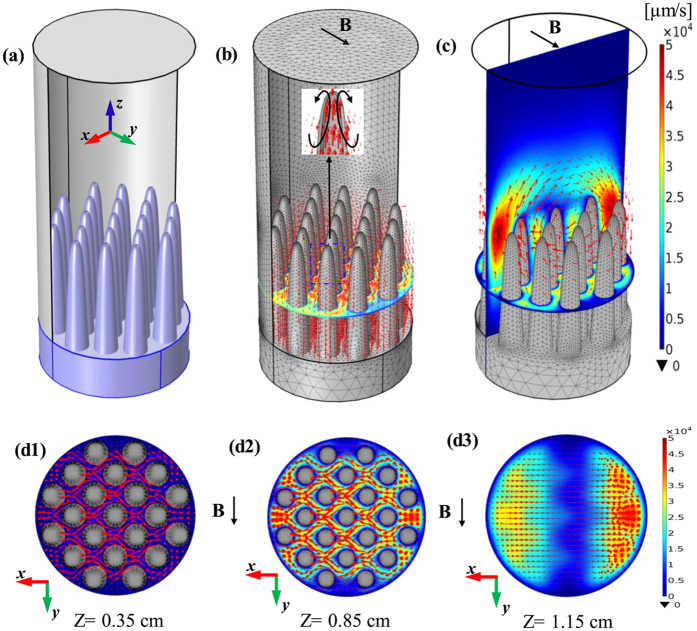
Numerical simulation for the TE magnetic effects in the directionally solidified Al-based alloys under a 0.5 T transverse magnetic field: (**a**) Geometry of computation domain; (**b**) computed TE current; (**c**) computed TE magnetic convection; (**d**) direction and magnitude of the computed TE magnetic convection in the x-y plane at different positions in the mushy zone.

**Figure 9:**
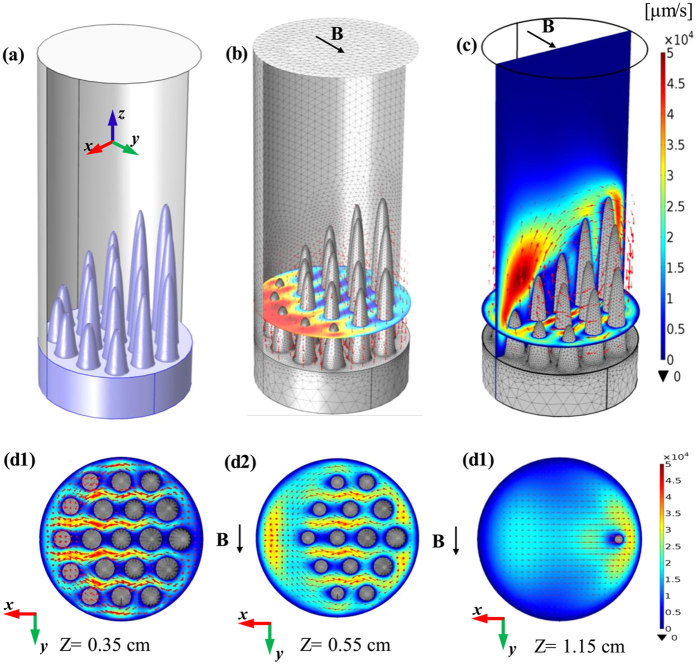
Numerical simulation for the TE magnetic effects in the directionally solidified Al-based alloys near the sloping solid/liquid interface during directional solidification under a transverse magnetic field of 0.5 T: (**a**) Geometry of computation domain; (**b**) computed TE current; (**c**) computed TE magnetic convection; (**d**) direction and magnitude of the computed TE magnetic convection on the x-y plane at different positions in the mushy zone.

**Figure 10:**
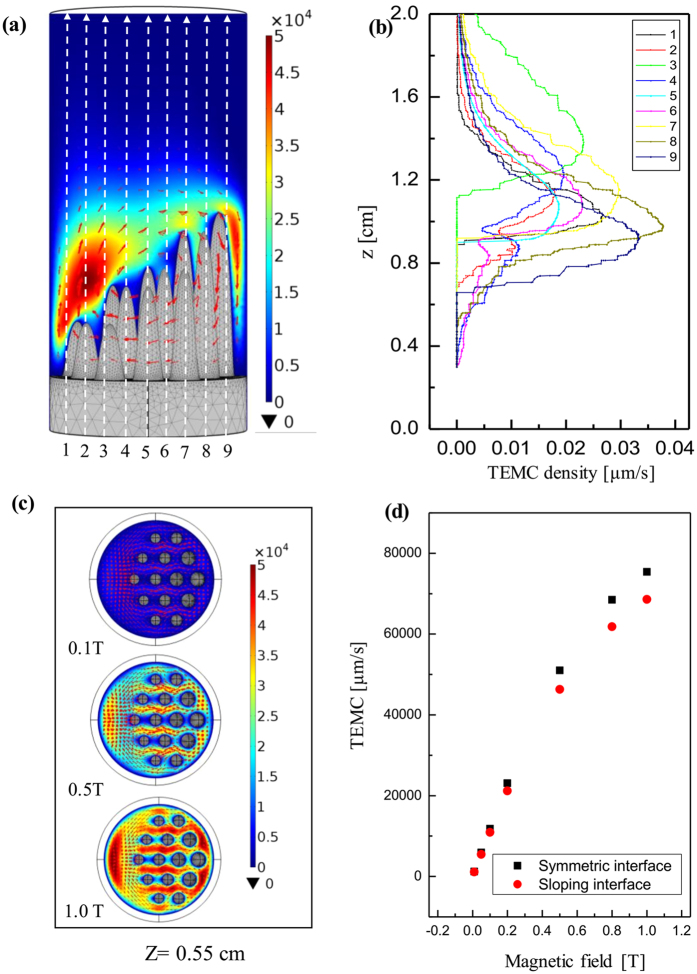
Distribution of the computed TE magnetic convection as a function of the magnetic field during directional solidification of the Al-based alloys under a transverse magnetic field: (**a**) Distribution of the TE magnetic convection on the x-z plane under a magnetic field of 0.5 T; (**b**) profile with different lines as shown in Fig. (**a**); (**c**) distribution of the TE magnetic convection on the x-z plane at 0.55 cm from the initial solidification interface under various magnetic fields; (**d**) the maximum value of the computed TE magnetic convection for the symmetric and sloping interfaces as a function of the magnetic field intensity.

Both Eqs (2) and (3) are valid in the liquid and solid. Another complementary term, 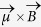 (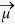 is the fluid velocity field and 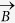 is the applied magnetic field), will be added to Eq. (2) when the magnetic field is present. Eqs (2) and (3) are solved in terms of the electric scalar potential *V* in the liquid and solid. More details on the equations and corresponding boundary conditions can be found in ref. 17. With a fixed and prescribed solid/liquid interface, this system is solved both in the liquid and solid phases simultaneously by a commercial finite element code COMSOL Multiphysics. It should be mentioned that a given interface is used as a heuristic model and qualitatively illustrates how the TE current and TE magnetic convection are generated. Figures 8,9 and 10 show the numerical simulation for the TE magnetic effects in directionally solidified Al-based alloys under a transverse magnetic field. The physical properties of the Al-based alloys used in the numerical simulation are given in Table 2. Figures 8(a) and 9(a) show the geometry of the computation domain of regular and sloping solid/liquid interfaces, respectively. The corresponding typical computed TE currents in the liquid near the solid/liquid interface are displayed in Figs 8(b) and 9(b), respectively. One can notice that the TE current forms the circuits along the cell and a maximum value of its density exists in the mushy region. Figures 8(c) and 9(c), respectively, show the general 3D view of the computed TE magnetic convection under a transverse magnetic field of 0.5 T and a temperature gradient of 60 K/cm. For a better observation, the corresponding 2D views seen from the positive z-axis at various positions are given in Figs 8(d) and 9(d). Note that the TE magnetic convection is almost unidirectional in the mushy zone. Furthermore, the distribution of the TE magnetic convection in the *x-z* plane under a 0.5 T transverse magnetic field is plotted in Fig. 10(a), which illustrates that the TE magnetic convection mainly occurs in the mushy zone (see Fig. 10(b)). Figure 10(c) shows the 2D view seen from the positive *z-*axis at a position of 0.55 cm from the solid phase under various magnetic fields (i.e., 0.1 T, 0.5 T and 1.0 T). The convection flows from the right side to the left side and its intensity increases with increasing magnetic field when the applied magnetic field is less than 1.0 T. Figure 10(d) shows the TE magnetic convection as a function of the magnetic field.

## Discussions

**Figure 11:**
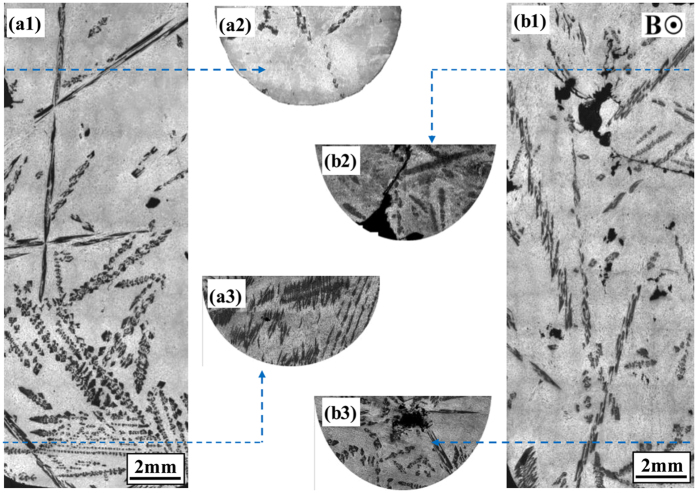
The effect of a static transverse magnetic field of 0.5 T on the structures in solidified Al-12 wt.%Ni alloys at a cooling rate of 2 K/min: (a1)-(a3) Longitudinal structure without the magnetic field and corresponding transverse structure at the bottom and tip of the sample; (b1)-(b3) longitudinal structures with the magnetic field and corresponding transverse structures at the bottom and tip of the sample.

To investigate the formation mechanism of the macrosegregation of the primary phases during directional solidification under the magnetic fields, the effect of the static magnetic field on the distribution of the primary phase during volume solidification is studied. Figure 11 shows the structure in solidified Al-12wt.%Ni alloys at a cooling rate of 2 K/min without and with a 0.5 T magnetic field. In the case of no magnetic field, the macrosegregation of the primary Al_3_Ni phase occurs (see Fig. 11(a)). Such macrosegregation is suppressed when a magnetic field is applied (see Fig. 11(b)). As the heavier Ni species migrates down due to the gravitation force, macrosegregation of the primary Al_3_Ni phase will form without a magnetic field. When a magnetic field is applied, the migration of the heavier Ni species is hindered. Therefore, macrosegregation of the primary Al_3_Ni phase disappears under a magnetic field. In the present work, the alloys are directionally solidified by using a vertical Bridgman setup with the melt above the crystal. Generally, for the vertical Bridgman crystal growth of alloys with the melt above the crystal, the heavier species migrates down due to the gravitation force. From the present experiments and the corresponding numerical simulations, it can be deduced that the TE magnetic convection should be responsible for the macrosegregation of the primary phases during directional solidification under the magnetic fields. This is well consistent with the previous investigations relevance between fluid flow and macrosegregation formation^18^,^19^,^20^,^21^,^22^,^23^,^24^,^25^,^26^.

Furthermore, to validate the numerical simulation results, a theoretical estimation has been done. The amplitude of the TE magnetic convection during the directional solidification of the Al-Cu alloys has been studied^14^. The results show that the fluid velocity originally increases as *B*^1/2^ and subsequently decreases as *B*^−1^. There is a maximum velocity, which corresponds to the condition where the TE magnetic convection is balanced with viscous friction and electromagnetic braking. In this case, *B*_max_ and the corresponding *u*_max_ are calculated by the following equation:


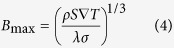



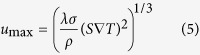


where *σ, S, λ* and *ρ* denote the electrical conductivity, the absolute TE power of the conducting medium, the scale and the density, respectively. For interdendritic TE magnetic convection, *λ* has an order of magnitude of 10 μm. The estimation results reveal that *B*_max_ is 0.65 T and the corresponding *u*_max_ is 0.7 × 10^−2^ m/s. This is in rough agreement with the numerical simulation results.

Thus, at the initial growth of the samples, the forced TE magnetic convection generates a rigorous solute exchange between the mushy zone and the bulk melt and encourages the primary phases to continuously precipitate and segregate. As the solute content in the bulk melt gradually approaches the eutectic point, precipitation of the primary phases is profoundly reduced. Moreover, the forced TE magnetic convection during directional solidification under a transverse magnetic field is unidirectional. The unidirectional TE magnetic convection and the corresponding recirculation loops will cause the heavier solute to move along the direction perpendicular to the magnetic field. As a consequence, a sloping solid/liquid interface will form during directional solidification under a transverse magnetic field.

**Figure 12:**
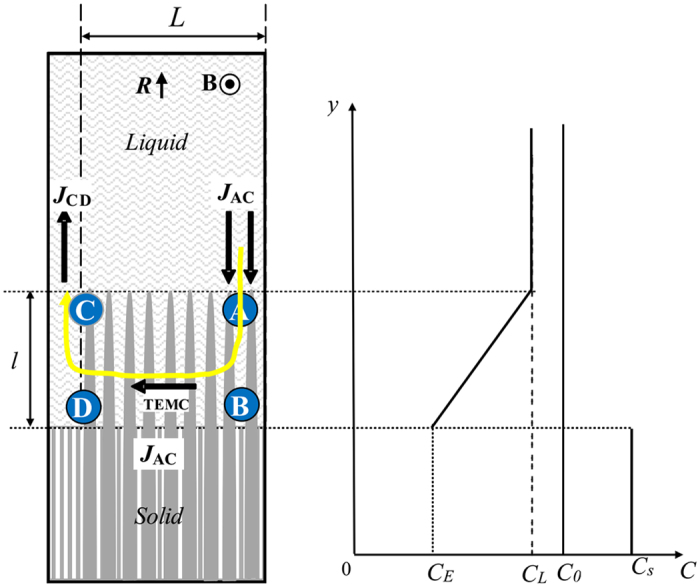
Sketch of the geometry and the control volume (ABCD) under the TE magnetic convection used for the model during directional solidification under a transverse magnetic field.

Furthermore, a model is built to analyze the effect of a transverse magnetic field on the macrosegregation during directional solidification. The effective segregation coefficient (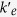) and the macrosegregation are determined through an integral control volume approach, as shown in Fig. 12. The control volume includes part of the solid/liquid interface exposed to the TE magnetic convection and the mushy zone having length *l* attached to it. The boundaries BD and AC have the length *L*. BD extends into the growing crystal. AC is immediately outside the mushy zone. Boundary AB is located at the crucible wall, preventing solute transfer. At the end of the region of flow, CD cuts across the mushy zone. For the sake of simplify, we suppose that the distribution of the solute between dendrites in the mushy zone is linear and the solutes at the tip and bottom of the dendrite are, respective, *C*_*E*_ and *C*_*l*_.

The volumetric outflow from the control volume through CD is equal to


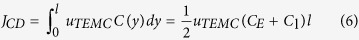


where *u*_*TEMC*_ is the velocity of the TE magnetic convection. The outflow of the solute incorporated in the solid *J*_*BD*_ is equal to


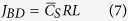


where 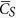 is the solid solubility and *R* is the growth speed. If we neglect the density change during solidification and assume that the melt is incompressible, the solute concentration at AC is taken as being uniform and equal to *C*_*L*_. As a result, the total solute inflow in the control volume is equal to:


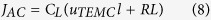


According to the steady state conservation of solute in the control volume, the solute inflow is equal to the solute outflow:


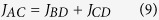


Substitution of Eqs (6), (7) and (8) into Eq. (9) and rearrangement yield an explicit equation for 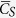



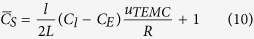


When the volume of the melt is large enough, *C*_*l*_ = *C*_*O*_; 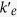 is


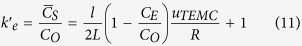


In our previous work^14^, the value of the TE magnetic convection as a function of the magnetic field was studied and can be written as


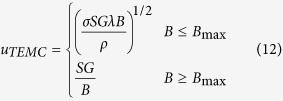


where *B*_max_ is the critical value when the TE magnetic convection reaches the maximal value, *G* is the temperature gradient, *S* is the TE power and *λ* is the characteristic length. Substitution of Eq. (12) into Eq. (11) and rearrangement yield an explicit equation for 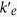



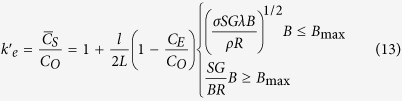


In this work, for Al-based hypereutectic alloys (i.e., Al-40 wt.%Cu and Al-12 wt.%Ni alloys), 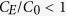. According to Eq. (13), with the increase of the temperature gradient (*G*) and decrease of the growth speed (*R*), the value of 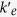 will increase gradually.

Moreover, from the numerical and estimation results, when the applied magnetic field is less than 0.5 T, the value of the TE magnetic convection in the Al-based alloys increases as the magnetic field increases. According to Eq. (11), the value of 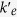 increases as the magnetic field increases when the magnetic field is less than 0.5 T. Thus, it can be concluded that the value of 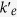 increases and the primary phase has a tendency to disappear with the increase of the magnetic field and temperature gradient and decrease of the growth speed (see Figs 1, 2, 3, 4, 5 and 6). The present work demonstrates that by solely applying a static magnetic field to directionally solidified alloys, macrosegregation can be driven as a result of the produced TE magnetic convection. This study not only presents a new approach to control the solute distribution in solidified alloys but also presents a new way to control forced flow in the solidification process.

## Conclusions

This work investigated the macrosegregation formation mechanism of the primary phases in four directionally solidified alloys (i.e., Al-Cu, Al-Si, Al-Ni and Zn-Cu alloys) under a static transverse magnetic field. The experimental results showed that significant macrosegregation of the primary phases (i.e., α-Al, Al_2_Cu, Al_3_Ni and Zn_5_Cu phases) occurred in these alloys at the initial solidification stage of the samples. The macrosegregation was enhanced with the increase of the magnetic field and temperature gradient and decrease of the growth speed. The numerical simulations revealed that a unidirectional TE magnetic convection formed in the mushy zone as a consequence of the interaction between the magnetic field and TE current. The value of the TE magnetic convection increased with the increase of the magnetic field when the applied magnetic field was less than 0.5 T. A model was proposed to explain the peculiar macrosegregation phenomenon during directional solidification under a transverse magnetic field. The macrosegregation of the primary phase was mainly related with the temperature gradient, the magnetic field and the growth speed. With the increase of the magnetic field and temperature gradient and decrease of the growth speed, the macrosegregation of the primary phase increaseed. This findings was in good agreement with the experimental results. Therefore, the formation of the macrosegregation during directional solidification under a transverse magnetic field should be attributed to the TE magnetic convection. The knowledge gained from this study shows a potential approach to control the solute distribution by applying a static magnetic field through a generated forced TE magnetic convection.

## Additional Information

**How to cite this article**: Xi, L. *et al*. Formation mechanism of axial macrosegregation of primary phases induced by a static magnetic field during directional solidification. *Sci. Rep.* **7**, 45834; doi: 10.1038/srep45834 (2017).

**Publisher's note:** Springer Nature remains neutral with regard to jurisdictional claims in published maps and institutional affiliations.

## Figures and Tables

**Table 1:** Area percentage of the primary phases in the directionally solidified Al-12 wt.%Ni alloy (%) (*R* = 3 μm/s and *G* = 60 K/cm).

Growth length (cm)	0–1.0	1.0–2.0	3.0–4.0	4.0–5.0
0 T	9.8 ± 0.2	9.6 ± 0.2	9.3 ± 0.3	9.2 ± 0.2
0.1 T	34.2 ± 0.3	28.3 ± 0.1	10.2 ± 0.5	0
0.5 T	42.2 ± 0.2	25.8 ± 0.3	5.2 ± 0.3	0

**Table 2:** Physical properties and parameters used in numerical simulation.

Name and symbol	Unit	Solid	Liquid
Absolute thermoelectric power (*S*)	V/K	−1.5 × 10^−6^	−2.25 × 10^−6^
Electrical conductivity (σ)	(Ω m)^−1^	7.9 × 10^7^	4.0 × 10^6^
Dynamic viscosity (μ)	Pa s	—	2.9 × 10^−3^
Density (ρ)	Kg/m^3^	2.7 × 10^3^	2.4 × 10^3^
Thermal conductivity (λ)	W/mK	150	90
